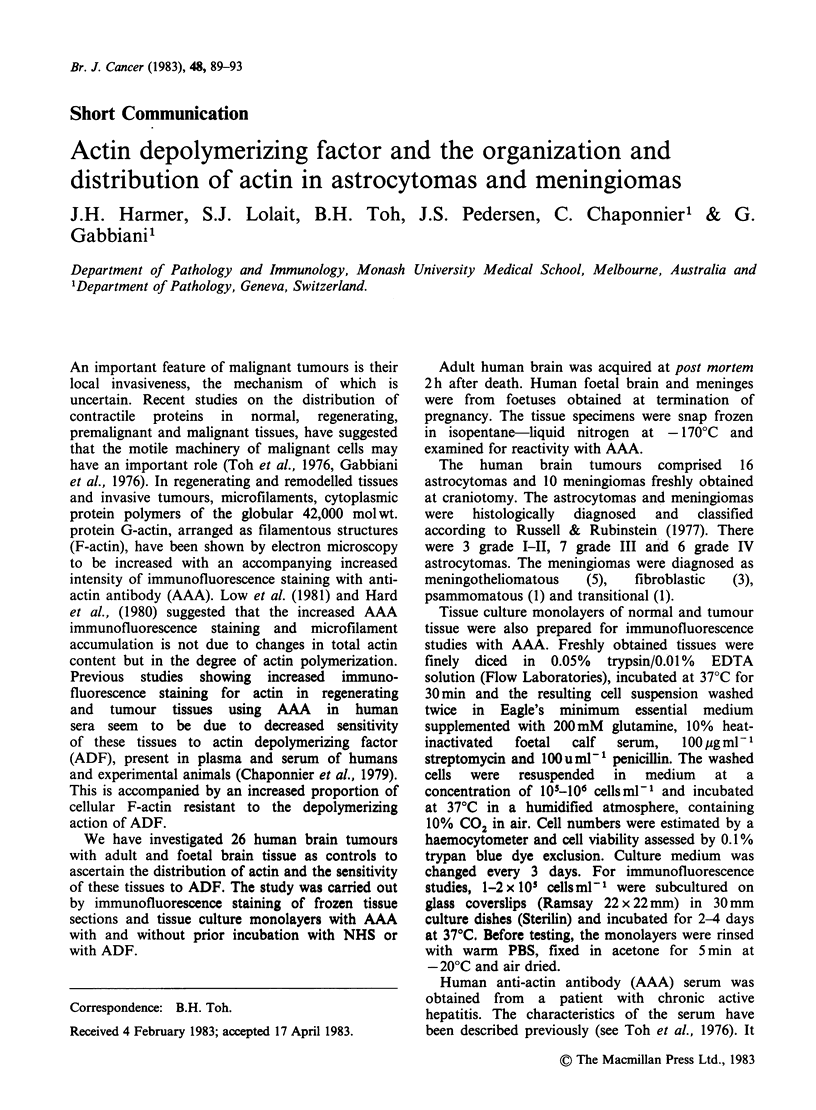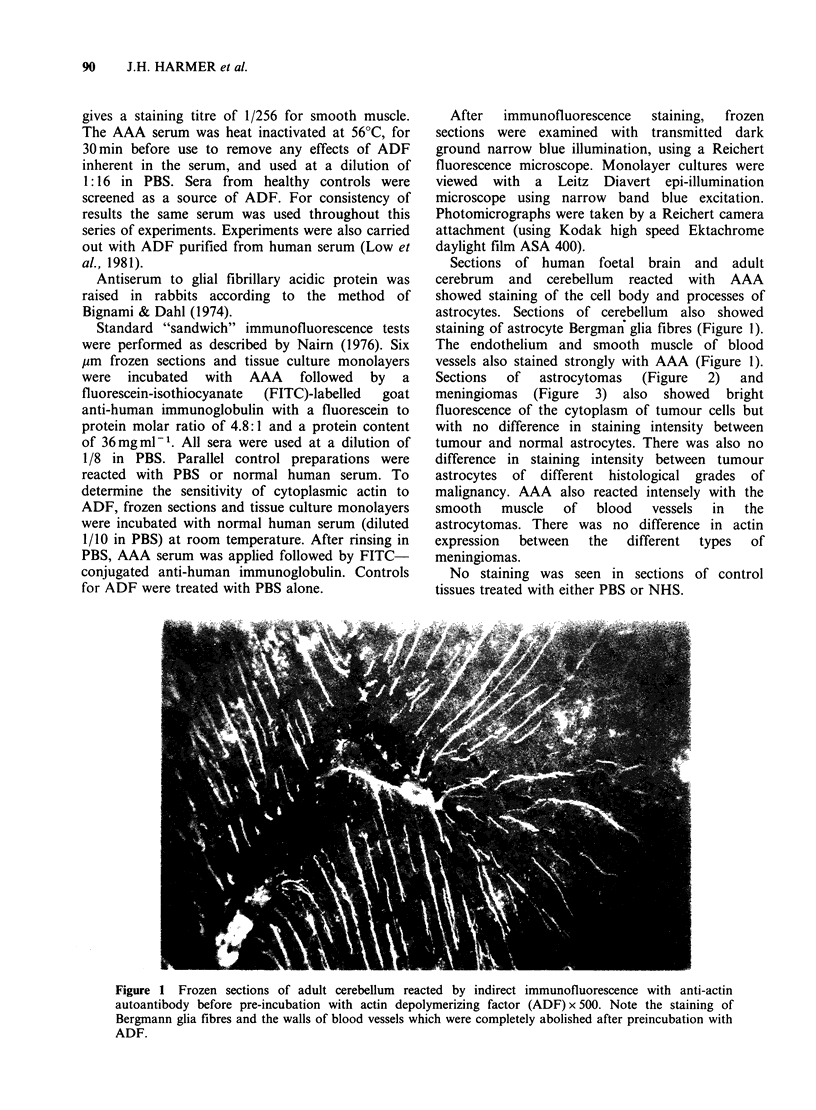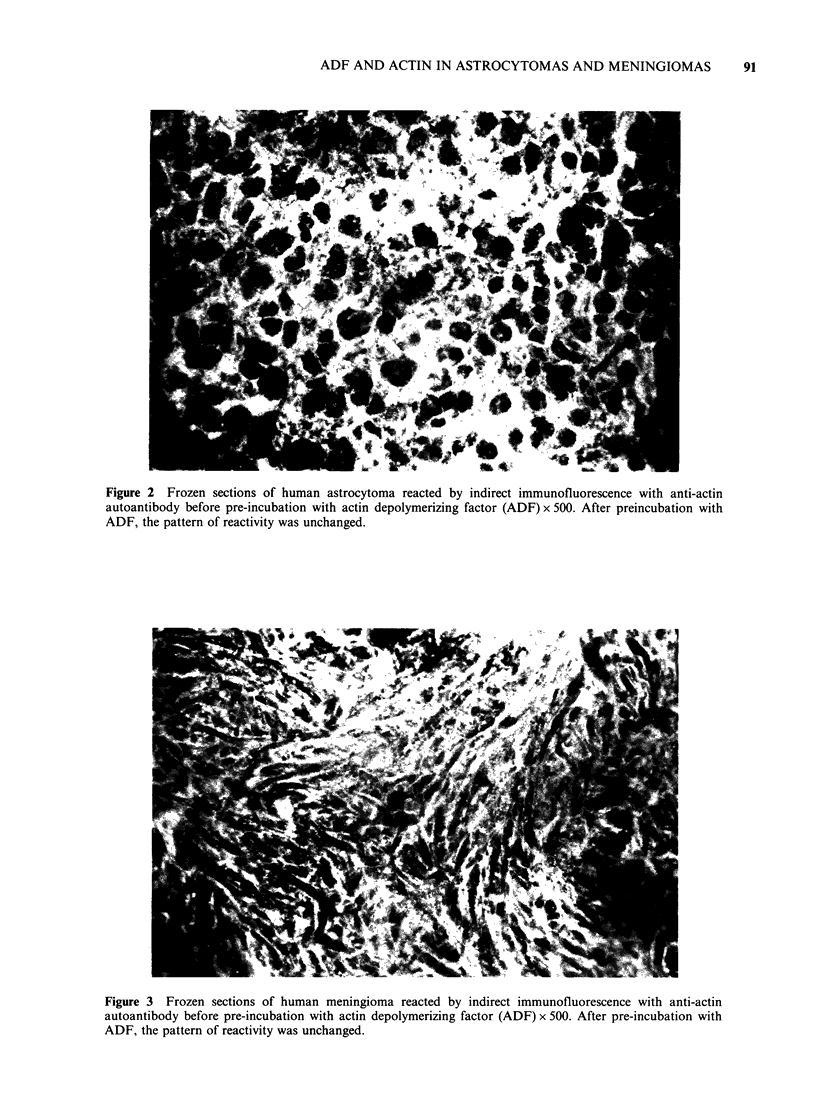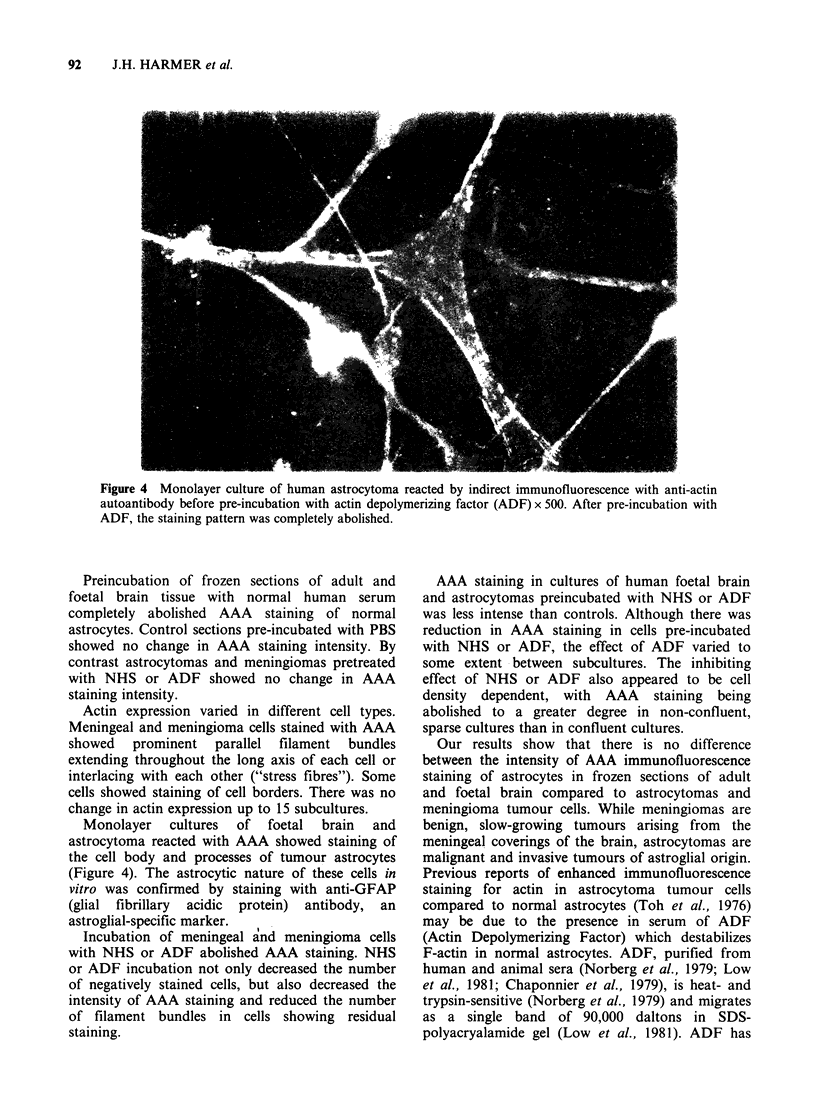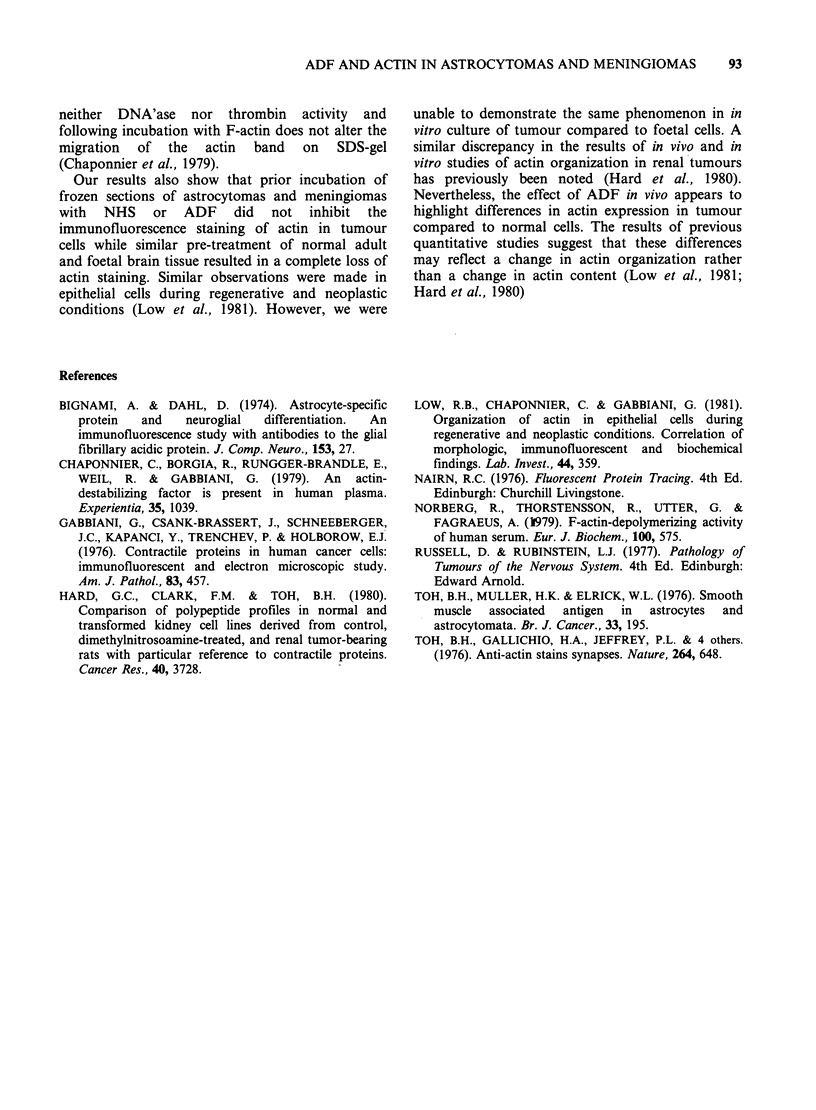# Actin depolymerizing factor and the organization and distribution of actin in astrocytomas and meningiomas.

**DOI:** 10.1038/bjc.1983.161

**Published:** 1983-07

**Authors:** J. H. Harmer, S. J. Lolait, B. H. Toh, J. S. Pedersen, C. Chaponnier, G. Gabbiani

## Abstract

**Images:**


*Br. J. Cancer* (1983), **48**, 89–93

### Short Communication

### Actin depolymerizing factor and the organization and distribution of actin in astrocytomas and meningiomas

J.H. Harmer, S.J. Lolait, B.H. Toh, J.S. Pedersen, C. Chaponnier[1] & G. Gabbiani[1]

*Department of Pathology and Immunology, Monash University Medical School, Melbourne, Australia and [1]Department of Pathology, Geneva, Switzerland.*

An important feature of malignant tumours is their local invasiveness, the mechanism of which is uncertain. Recent studies on the distribution of contractile proteins in normal, regenerating, premalignant and malignant tissues, have suggested that the motile machinery of malignant cells may have an important role (Toh *et al.*, 1976, Gabbiani *et al.*, 1976). In regenerating and remodelled tissues and invasive tumours, microfilaments, cytoplasmic protein polymers of the globular 42,000 mol wt. protein G-actin, arranged as filamentous structures (F-actin), have been shown by electron microscopy to be increased with an accompanying increased intensity of immunofluorescence staining with anti-actin antibody (AAA). Low *et al.* (1981) and Hard *et al.*, (1980) suggested that the increased AAA immunofluorescence staining and microfilament accumulation is not due to changes in total actin content but in the degree of actin polymerization. Previous studies showing increased immunofluorescence staining for actin in regenerating and tumour tissues using AAA in human sera seem to be due to decreased sensitivity of these tissues to actin depolymerizing factor (ADF), present in plasma and serum of humans and experimental animals (Chaponnier *et al.*, 1979). This is accompanied by an increased proportion of cellular F-actin resistant to the depolymerizing action of ADF.

We have investigated 26 human brain tumours with adult and foetal brain tissue as controls to ascertain the distribution of actin and the sensitivity of these tissues to ADF. The study was carried out by immunofluorescence staining of frozen tissue sections and tissue culture monolayers with AAA with and without prior incubation with NHS or with ADF.

Correspondence: B.H. Toh.

Received 4 February 1983; accepted 17 April 1983.

Adult human brain was acquired at *post mortem* 2 h after death. Human foetal brain and meninges were from foetuses obtained at termination of pregnancy. The tissue specimens were snap frozen in isopentane—liquid nitrogen at $-170°C$ and examined for reactivity with AAA.

The human brain tumours comprised 16 astrocytomas and 10 meningiomas freshly obtained at craniotomy. The astrocytomas and meningiomas were histologically diagnosed and classified according to Russell & Rubinstein (1977). There were 3 grade I–II, 7 grade III and 6 grade IV astrocytomas. The meningiomas were diagnosed as meningotheliomatous (5), fibroblastic (3), psammomatous (1) and transitional (1).

Tissue culture monolayers of normal and tumour tissue were also prepared for immunofluorescence studies with AAA. Freshly obtained tissues were finely diced in 0.05% trypsin/0.01% EDTA solution (Flow Laboratories), incubated at 37°C for 30 min and the resulting cell suspension washed twice in Eagle's minimum essential medium supplemented with 200 mM glutamine, 10% heat-inactivated foetal calf serum, $100\,\mu g\,ml^{-1}$ streptomycin and $100\,u\,ml^{-1}$ penicillin. The washed cells were resuspended in medium at a concentration of $10^5$–$10^6$ cells $ml^{-1}$ and incubated at 37°C in a humidified atmosphere, containing 10% $CO_2$ in air. Cell numbers were estimated by a haemocytometer and cell viability assessed by 0.1% trypan blue dye exclusion. Culture medium was changed every 3 days. For immunofluorescence studies, $1$–$2\times10^5$ cells $ml^{-1}$ were subcultured on glass coverslips (Ramsay 22 x 22 mm) in 30 mm culture dishes (Sterilin) and incubated for 2–4 days at 37°C. Before testing, the monolayers were rinsed with warm PBS, fixed in acetone for 5 min at $-20°C$ and air dried.

Human anti-actin antibody (AAA) serum was obtained from a patient with chronic active hepatitis. The characteristics of the serum have been described previously (see Toh *et al.*, 1976). It

© The Macmillan Press Ltd., 1983

gives a staining titre of 1/256 for smooth muscle. The AAA serum was heat inactivated at 56°C, for 30 min before use to remove any effects of ADF inherent in the serum, and used at a dilution of 1:16 in PBS. Sera from healthy controls were screened as a source of ADF. For consistency of results the same serum was used throughout this series of experiments. Experiments were also carried out with ADF purified from human serum (Low *et al.*, 1981).

Antiserum to glial fibrillary acidic protein was raised in rabbits according to the method of Bignami & Dahl (1974).

Standard "sandwich" immunofluorescence tests were performed as described by Nairn (1976). Six $\mu$m frozen sections and tissue culture monolayers were incubated with AAA followed by a fluorescein-isothiocyanate (FITC)-labelled goat anti-human immunoglobulin with a fluorescein to protein molar ratio of 4.8:1 and a protein content of 36 mg ml$^{-1}$. All sera were used at a dilution of 1/8 in PBS. Parallel control preparations were reacted with PBS or normal human serum. To determine the sensitivity of cytoplasmic actin to ADF, frozen sections and tissue culture monolayers were incubated with normal human serum (diluted 1/10 in PBS) at room temperature. After rinsing in PBS, AAA serum was applied followed by FITC—conjugated anti-human immunoglobulin. Controls for ADF were treated with PBS alone.

After immunofluorescence staining, frozen sections were examined with transmitted dark ground narrow blue illumination, using a Reichert fluorescence microscope. Monolayer cultures were viewed with a Leitz Diavert epi-illumination microscope using narrow band blue excitation. Photomicrographs were taken by a Reichert camera attachment (using Kodak high speed Ektachrome daylight film ASA 400).

Sections of human foetal brain and adult cerebrum and cerebellum reacted with AAA showed staining of the cell body and processes of astrocytes. Sections of cerebellum also showed staining of astrocyte Bergman glia fibres (Figure 1). The endothelium and smooth muscle of blood vessels also stained strongly with AAA (Figure 1). Sections of astrocytomas (Figure 2) and meningiomas (Figure 3) also showed bright fluorescence of the cytoplasm of tumour cells but with no difference in staining intensity between tumour and normal astrocytes. There was also no difference in staining intensity between tumour astrocytes of different histological grades of malignancy. AAA also reacted intensely with the smooth muscle of blood vessels in the astrocytomas. There was no difference in actin expression between the different types of meningiomas.

No staining was seen in sections of control tissues treated with either PBS or NHS.

**Figure 1** Frozen sections of adult cerebellum reacted by indirect immunofluorescence with anti-actin autoantibody before pre-incubation with actin depolymerizing factor (ADF) × 500. Note the staining of Bergmann glia fibres and the walls of blood vessels which were completely abolished after preincubation with ADF.

**Figure 2** Frozen sections of human astrocytoma reacted by indirect immunofluorescence with anti-actin autoantibody before pre-incubation with actin depolymerizing factor (ADF) × 500. After preincubation with ADF, the pattern of reactivity was unchanged.

**Figure 3** Frozen sections of human meningioma reacted by indirect immunofluorescence with anti-actin autoantibody before pre-incubation with actin depolymerizing factor (ADF) × 500. After pre-incubation with ADF, the pattern of reactivity was unchanged.

**Figure 4** Monolayer culture of human astrocytoma reacted by indirect immunofluorescence with anti-actin autoantibody before pre-incubation with actin depolymerizing factor (ADF) × 500. After pre-incubation with ADF, the staining pattern was completely abolished.

Preincubation of frozen sections of adult and foetal brain tissue with normal human serum completely abolished AAA staining of normal astrocytes. Control sections pre-incubated with PBS showed no change in AAA staining intensity. By contrast astrocytomas and meningiomas pretreated with NHS or ADF showed no change in AAA staining intensity.

Actin expression varied in different cell types. Meningeal and meningioma cells stained with AAA showed prominent parallel filament bundles extending throughout the long axis of each cell or interlacing with each other ("stress fibres"). Some cells showed staining of cell borders. There was no change in actin expression up to 15 subcultures.

Monolayer cultures of foetal brain and astrocytoma reacted with AAA showed staining of the cell body and processes of tumour astrocytes (Figure 4). The astrocytic nature of these cells *in vitro* was confirmed by staining with anti-GFAP (glial fibrillary acidic protein) antibody, an astroglial-specific marker.

Incubation of meningeal and meningioma cells with NHS or ADF abolished AAA staining. NHS or ADF incubation not only decreased the number of negatively stained cells, but also decreased the intensity of AAA staining and reduced the number of filament bundles in cells showing residual staining.

AAA staining in cultures of human foetal brain and astrocytomas preincubated with NHS or ADF was less intense than controls. Although there was reduction in AAA staining in cells pre-incubated with NHS or ADF, the effect of ADF varied to some extent between subcultures. The inhibiting effect of NHS or ADF also appeared to be cell density dependent, with AAA staining being abolished to a greater degree in non-confluent, sparse cultures than in confluent cultures.

Our results show that there is no difference between the intensity of AAA immunofluorescence staining of astrocytes in frozen sections of adult and foetal brain compared to astrocytomas and meningioma tumour cells. While meningiomas are benign, slow-growing tumours arising from the meningeal coverings of the brain, astrocytomas are malignant and invasive tumours of astroglial origin. Previous reports of enhanced immunofluorescence staining for actin in astrocytoma tumour cells compared to normal astrocytes (Toh *et al.*, 1976) may be due to the presence in serum of ADF (Actin Depolymerizing Factor) which destabilizes F-actin in normal astrocytes. ADF, purified from human and animal sera (Norberg *et al.*, 1979; Low *et al.*, 1981; Chaponnier *et al.*, 1979), is heat- and trypsin-sensitive (Norberg *et al.*, 1979) and migrates as a single band of 90,000 daltons in SDS-polyacryalamide gel (Low *et al.*, 1981). ADF has neither DNA'ase nor thrombin activity and following incubation with F-actin does not alter the migration of the actin band on SDS-gel (Chaponnier *et al.*, 1979).

Our results also show that prior incubation of frozen sections of astrocytomas and meningiomas with NHS or ADF did not inhibit the immunofluorescence staining of actin in tumour cells while similar pre-treatment of normal adult and foetal brain tissue resulted in a complete loss of actin staining. Similar observations were made in epithelial cells during regenerative and neoplastic conditions (Low *et al.*, 1981). However, we were unable to demonstrate the same phenomenon in *in vitro* culture of tumour compared to foetal cells. A similar discrepancy in the results of *in vivo* and *in vitro* studies of actin organization in renal tumours has previously been noted (Hard *et al.*, 1980). Nevertheless, the effect of ADF *in vivo* appears to highlight differences in actin expression in tumour compared to normal cells. The results of previous quantitative studies suggest that these differences may reflect a change in actin organization rather than a change in actin content (Low *et al.*, 1981; Hard *et al.*, 1980)

### References

BIGNAMI, A. & DAHL, D. (1974). Astrocyte-specific protein and neuroglial differentiation. An immunofluorescence study with antibodies to the glial fibrillary acidic protein. *J. Comp. Neuro.*, **153**, 27.

CHAPONNIER, C., BORGIA, R., RUNGGER-BRANDLE, E., WEIL, R. & GABBIANI, G. (1979). An actin-destabilizing factor is present in human plasma. *Experientia*, **35**, 1039.

GABBIANI, G., CSANK-BRASSERT, J., SCHNEEBERGER, J.C., KAPANCI, Y., TRENCHEV, P. & HOLBOROW, E.J. (1976). Contractile proteins in human cancer cells: immunofluorescent and electron microscopic study. *Am. J. Pathol.*, **83**, 457.

HARD, G.C., CLARK, F.M. & TOH, B.H. (1980). Comparison of polypeptide profiles in normal and transformed kidney cell lines derived from control, dimethylnitrosoamine-treated, and renal tumor-bearing rats with particular reference to contractile proteins. *Cancer Res.*, **40**, 3728.

LOW, R.B., CHAPONNIER, C. & GABBIANI, G. (1981). Organization of actin in epithelial cells during regenerative and neoplastic conditions. Correlation of morphologic, immunofluorescent and biochemical findings. *Lab. Invest.*, **44**, 359.

NAIRN, R.C. (1976). *Fluorescent Protein Tracing*. 4th Ed. Edinburgh: Churchill Livingstone.

NORBERG, R., THORSTENSSON, R., UTTER, G. & FAGRAEUS, A. (1979). F-actin-depolymerizing activity of human serum. *Eur. J. Biochem.*, **100**, 575.

RUSSELL, D. & RUBINSTEIN, L.J. (1977). *Pathology of Tumours of the Nervous System*. 4th Ed. Edinburgh: Edward Arnold.

TOH, B.H., MULLER, H.K. & ELRICK, W.L. (1976). Smooth muscle associated antigen in astrocytes and astrocytomata. *Br. J. Cancer.*, **33**, 195.

TOH, B.H., GALLICHIO, H.A., JEFFREY, P.L. & 4 others. (1976). Anti-actin stains synapses. *Nature*, **264**, 648.